# Allostery-Driven Substrate Gating in the Chlorothalonil Dehalogenase from *Pseudomonas* sp. CTN-3

**DOI:** 10.3390/biology15010020

**Published:** 2025-12-22

**Authors:** Grayson Gerlich, Judith Klein-Seetharaman, Richard C. Holz

**Affiliations:** 1 Quantitative Biosciences and Engineering Program, Colorado School of Mines, Golden, CO 80401, USA; ggerlich@mines.edu; 2 Department of Chemistry, Colorado School of Mines, Golden, CO 80401, USA; 3 School of Molecular Sciences, Arizona State University, Phoenix, AZ 85004, USA

**Keywords:** dehalogenase, dechlorination, molecular dynamics, docking, zinc, metalloenzyme, bioremediation, chlorothalonil, enzyme catalysis, enzyme mechanism, chlorinated aromatic hydrocarbons

## Abstract

The catalytic mechanism of the Zn(II)-dependent chlorothalonil dehalogenase from *Pseudomonas* sp. CTN-3 (Chd) was examined using molecular dynamics (MD) simulations, Bayesian network analysis, and Markov state model analysis to quantify its motions. Chd selectively substitutes an aromatic chlorine-carbon bond in chlorothalonil (TPN; 2,4,5,6-tetrachloroisophtalonitrile) with an aromatic alcohol (4-hydroxytrichloro-isophthalonitrile; 4-OH-TPN). It is a homodimer with two solvent-accessible channels in each monomer, which are proposed to provide different routes for substrate and products to access/leave the catalytic Zn(II) site. Based on MD simulations, Chd exhibits allosteric behavior wherein a “Y”-shaped substrate channel exhibits a “flip flop” mechanism, where the “right” substrate channel opens to allow TPN to enter, after which it closes, followed by the “left” channel opening. The “right” channel then reopens, likely to allow the product, 4-OH-TPN, to leave the active site, but this reopening of the right channel drives the “left” channel to close. Coupled with the substrate channels alternately opening and closing, a corresponding possible Cl^−^ channel opens and closes. Although the dynamics of this process are fast, Chd needs to overcome a 5 kT free-energy barrier for this transition and to relax after opening. Additionally, exposed “wing” residues, hydrophilic residues at the ends of protruding α-helices, act as allosteric indicators, signaling the complex allosteric motions required to open the substrate channel. We propose, for the first time, a dynamic mechanism that drives substrate binding and product release, providing new insight into Chd’s catalytic mechanism.

## Simple Summary

A structural dynamics study of Chd using molecular dynamics simulations, Bayesian network analysis, and Markov state model analysis to quantify observed motions. Chd exhibits allosteric behavior wherein a “Y”-shaped substrate channel exhibits a “flip flop” mechanism, opening only one side of a dimer to substrate at any time. These data link allostery between monomers and a stable but highly dynamic protein that leverages molecular motions to enable catalysis, providing new insight into the catalytic mechanism of Chd.

## 1. Introduction

Modern production of halogenated aromatic compounds has generated an arsenal of useful products for commercial and industrial applications, but the very properties that make these compounds so appealing also make them highly dangerous environmental contaminants [1,2]. These halogenated chemicals are stable, persistent in the environment, and often toxic to humans, wildlife, soil microbiota, and more, making bioremediation of these compounds an important goal [3,4,5,6]. Dehalogenation, in general, provides more soluble forms of these compounds that are less likely to bioaccumulate, more likely to degrade further and are, thus, less toxic. Enzymatic dehalogenation offers a bioremediation solution for halogenated compounds; however, a major impediment to understanding the biological role and the bioremediation uses of these enzymes is the lack of a detailed understanding of their catalytic mechanisms. The prevailing dogma is that biological dehalogenation reactions are catalyzed by oxidative, reductive, or thiolytic dehalogenation processes, but a lesser-known biological dehalogenation process exists that involves hydrolysis of a C–Cl bond. Hydrolytic dehalogenases represent a unique opportunity to create biomaterials with the ability to improve current bioremediation technologies [1,7,8,9,10].

One such hydrolytic dehalogenase is chlorothalonil dehalogenase from *Pseudomonas* sp. CTN-3 (Chd), a Zn(II)-dependent, homodimeric enzyme that hydrolytically dehalogenates chlorothalonil (TPN; 2,4,5,6-tetrachloroisophthalonitrile), a poly-chlorinated broad-spectrum fungicide, to its hydroxylated form (4-OH-TPN; 4-hydroxytrichloroisophthalonitrile) [11]. TPN is toxic to aquatic species, causes birth defects in birds and mammals, is strongly absorbed in soils, causing damage to soil microbiota, and is persistent on produce [5,12,13,14,15]. TPN can enter waterways and in areas with heavy TPN use, inhabitants are at risk of significant exposure and illness [15,16,17,18,19]. These factors, combined with EPA guidance suggesting TPN is a probable human carcinogen, makes remediation of TPN an urgent need for rural and urban communities alike [14].

**Figure 1:**
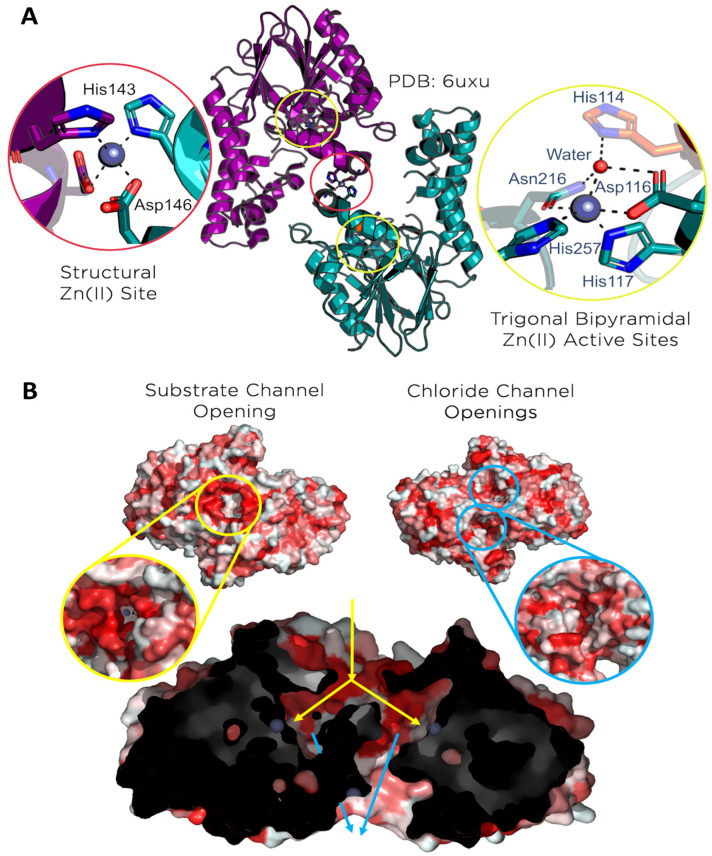
**The structure of Chd (PDB: 6UXU).** (**A**) A structural overview of the Chd dimer, highlighting the three Zn(II) centers present in the protein. Active site residues are represented by sticks, Zn(II) atoms are represented by gray spheres. (**B**) Views of the channels present in Chd. The channels are on opposing sides of the dimer. Residues are colored by increasing hydrophobicity (red → hydrophobic). Zn(II) atoms are visualized as gray spheres. The cut section of Chd shows the Y-shaped substrate channel and smaller secondary channels as they move through the protein.

The X-ray crystal structure of Chd (PDB: 6UXU) reveals a “head-to-tail” homodimer, with each monomer exhibiting an αββα-sandwich fold commonly associated with the metallo-β-lactamase (MBL) superfamily [20]. Two mononuclear Zn(II) centers, buried roughly 15 Å into each monomer, are catalytic sites, with a third mononuclear Zn(II) center located at the dimer interface, functioning as a structural site. The catalytic Zn(II) atoms display a slightly distorted trigonal bipyramid (TBP) geometry with His117, His257, Asp116, Asn216, and a water/hydroxide as ligands, while the structural Zn(II) atom displays a tetrahedral geometry with His143 and Asp146 ligands from each monomer (Figure 1A, Video S1). Of particular interest in the Chd structure are the prominent channels that allow access to the active sites. A Y-shaped channel originates on the dimer interface at a large opening (8.5 Å × 17.8 Å) ringed by the hydrophobic residues Leu28, Ile29, Leu307, and Phe310, that likely serve to recognize TPN (Figure 1B, Video S1). This channel branches ~9 Å under the protein’s surface, allowing access to both active sites. Two smaller channels, one per active site, exit the protein on the side opposite the substrate channel. Based on the size and hydrophilicity of these channels, they are hypothesized to help expel the chloride ions produced during catalytic turnover. Because Chd exhibits no more than 15% structural similarity to any other known proteins, the function and dynamics of these channels are unknown [20].

**Figure 2:**
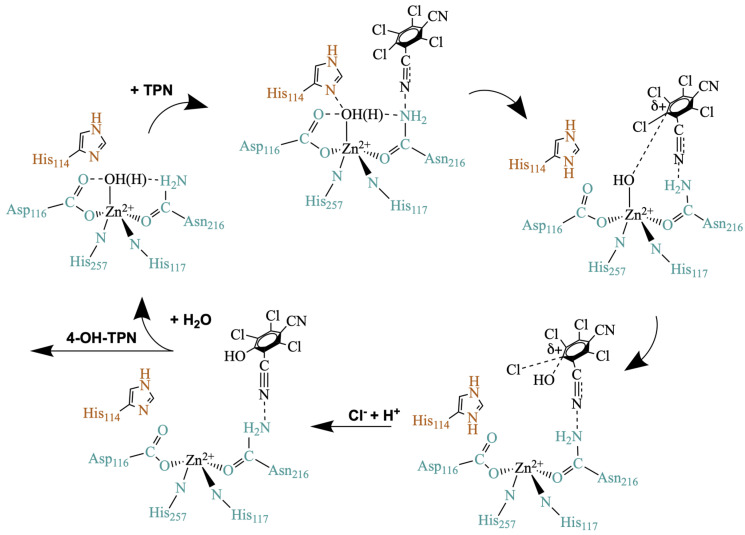
**Proposed mechanism for TPN hydrolysis by Chd**. Colors for metal binding residues and His114 are the same as used in Figure 1A.

Prior studies using EPR, mutagenesis, UV-Vis spectroscopy, docking, and molecular dynamics (MD) provided a proposed mechanism for Chd (Figure 2) [20,21,22]. This mechanism is consistent with TPN entering through the putative substrate channel, but without a crystal structure that shows a TPN binding conformation, it is difficult to confirm the validity of this mechanism. TPN binding was proposed to possibly elicit allosteric movements in Chd, as is often observed with multimeric proteins [23]. Reported herein are structural dynamics studies of Chd with molecular dynamics simulations, Bayesian network analysis, and Markov state model analysis. Chd exhibits allosteric behavior wherein the “Y”-shaped substrate channel exhibits a “flip flop” mechanism, opening only one dimer to substrate at any time. These data link allostery between monomers and a stable but highly dynamic protein that leverages molecular motions to enable catalysis, providing new insight into the catalytic mechanism of Chd.

## 2. Materials and Methods

### 2.1. All-Atomistic Molecular Dynamics (MD)

All simulations were performed in GROMACS 2024.4 [24,25,26]. The CHARMM36 force field was used and the system was solvated explicitly with the sTIP3P water model [27]. The preparation and execution of production MD runs largely followed established methods used for Chd [21]. Briefly, for all simulations, the time step was 2 fs, energies and coordinates were saved every 10 ps, and Particle Mesh Ewald long-range electrostatics were used. TPN was parameterized using the CGenFF webserver [27]. The simulation box was adjusted to a salt concentration of 0.15 M using the SPLIT method. Energy minimization was performed until the maximum system force was <10 kJ/(mol·nm). The system was then subjected to pressure and temperature equilibration with a modified Berendsen thermostat and C-rescale pressure coupling. For simulations where TPN was present, TPN’s placement in the active site was determined by docking studies using PLANTS as previously described, as well as Attracting Cavities as implemented in SwissDock (https://www.swissdock.ch/) [21,28,29]. Best-fit poses from both methods were selected and subjected to 100 ns production simulations and then compared. After short simulations, TPN oriented itself the same way in the active site pocket, indicating that long-timescale simulations were reasonably invariant to starting pose. This is consistent with prior work that indicated TPN lies within a flat substrate pocket that enables rotation for the generation of productive conformations [26]. Production MD runs on each ensemble were performed as triplicate 1 μs runs, for 3 μs total simulation time. Convergence was evaluated with the KMeans clustering algorithm as implemented in MDAnalysis (100 clusters using Cα atoms as features), as well as by analyzing the all-residue heavy-atom RMSD of the trajectory vs. the equilibrated structure using gmx rms, which measures how much a structure (or part of it) moves relative to a reference structure, such as the starting conformation or an equilibrated frame [30,31]. Simulations were only considered for analysis if the windows of the KMeans clusters rapidly reached a plateau Jenson–Shannon divergence near 0 and no significant disjoints were observed in the RMSD plot. Most simulations were performed on an in-house computer cluster with additional CPU time provided by NMRbox, a Biomedical Technology Research Resource (BTRR) that is supported by NIH grant P41GM111135 (NIGMS) [32].

### 2.2. Trajectory Analysis

Initial trajectory processing was performed using GROMACS. All output trajectories were centered and fit to eliminate translational and rotational motion. Unless stated otherwise, all analyses were performed on the full trajectory with no down sampling and the feature spaces were normalized. Clustering analysis of single ensembles and Markov state model (MSM) generation was performed using PyEMMA [33]. As PyEMMA is no longer actively maintained, a list of the Python package versions used to run PyEMMA is provided in the Supplementary Materials for reproducibility. The feature space for MSM analysis comprised all of the backbone and sidechain torsions, as well as the residue centers of mass (COMs) for all residues located within ~5 Å of a channel or active site cavity space (Figure S4). To check the validity of this selection, the relative entropy between the TPN-bound and non-TPN-bound ensembles was determined using PENSA as described below (Figure S5). Comparisons between principal component analysis (PCA) and time-lagged independent component analysis (TICA) as implemented in PyEMMA showed that PCA produced the best clustering results (Figure S1). The PCA model explained 95% of the model variance with 12 PCs, with a strong eigen separation after the first two components (Figure S6). Implied timescales (ITSs) were estimated using 100 clusters in the feature space generated by the top 2 PCs and 3 slow processes were resolved. The resulting MSM satisfied Markovianity within a 95% confidence interval with four metastable states, as assessed via the Chapman–Kolmogorow test, and was convergent at a minimum lag time of 30 ps. Perron Cluster (PCCA++) was used with method roeblitz-14 to cluster microstates into metastable states. Mean first passage time (MFPT) was calculated using PyEMMA’s built in MFPT function, and pathway fluxes were calculated using transition path theory (TPT) and flux modules. PCA and clustering were performed for the non-substrate bound ensemble and the resultant model was applied to the substrate-bound ensembles.

Clustering analysis, relative entropy, and multi-ensemble comparison was performed using PENSA. For reproducibility, a list of the Python package (3.13.5) versions used to run PENSA is provided in the Supplementary Materials. Briefly, for each input structure, all three trajectories were ensembled together. Examination of all-residue heavy-atom RMSD plots indicated that the first 2% of frames consistently contained non-equilibrium dynamics, so these frames were skipped during assembling. For clustering, an appropriate number of clusters were selected from the within-sum-of-squares (WSS) plot by visual inspection. Relative entropy was computed over the distribution of backbone and sidechain torsions and compared between ensembles using the Jenson–Shannon distance, as implemented in PENSA.

A Bayesian network analysis of simulations was performed using BaNDyT, using the same Python environment as used for PENSA [34]. Generation of input files for BaNDyT software was accomplished by calculating the total interaction energy of each residue with the rest of the protein using gmx energy, skipping the first 2% of frames as described above, with the resulting energies concatenated for ensembles. Following this, the molecular dynamics solver, the numerical engine that integrates Newton’s equations of motion for all atoms in the system to simulate their time evolution, was used with 50 iterations allowing convergence for all ensembles tested. For all ensembles, the number of orphaned nodes was under 2%, indicating a high degree of network coverage. For generating network visualizations in PyMol, custom python scripts were used to leverage the Cytoscape API [35].

### 2.3. Visual Analysis

Unless noted otherwise, structures were visualized and modified using the open-source distribution of PyMol. The following open-source PyMol scripts were used: color_h and pymol_viridis. Networks and directed acyclic graphs (DAGs) were visualized using Cytoscape (version 3.10.4) [36].

## 3. Results and Discussion

The prominent substrate channels and interior cavity spaces within Chd identified via X-ray crystallography, which allow access to both active site Zn^2+^ ions, are features that appear to dominate the structure and were hypothesized to play a role in catalysis [20]. Several important mechanistic questions arise from examination of these channels. First, if substrate accesses both active sites through a single hydrophobic substrate cavity, how does it choose which direction to go within the Y-shaped channel, i.e., which active site does it move towards for hydrolysis? Second, once it moves into an active site, what is the driving force to shuttle Cl^−^ out of the active site along with the product, i.e., do they both exit via the Y-shaped channel or does Cl^−^ exit through the hydrophobic channel? To gain insight into these questions, MD simulations were used to examine allosteric effects on substrate recognition and binding to Chd. All atomistic 3 μs simulation ensembles were generated for the structures of interest: WT Chd and WT Chd + TPN.

**Figure 3:**
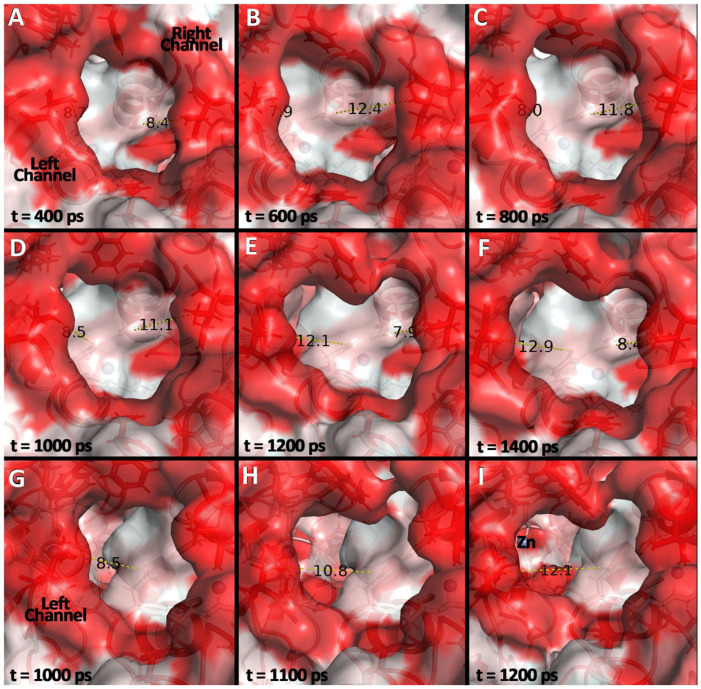
**Snapshots from a 1 us MD simulation of Chd.** (**A**–**F**): Each box shows the “main” substrate entrance of Chd, which branches into a Y-shaped channel with “left” and “right” branches. Chd is surface-colored by residue hydrophobicity (red → hydrophobic). The distances of Asn154 and Ile29 from both chains are visualized as a proxy for the channel width. (**A**) shows a transient state where both channels are “closed”. (**B**–**D**) show the “right” channel opening and relaxing (**E**) and (**F**) shows the “left” channel opening and reaching full aperture. (**G**–**I**): snapshots showing the “left” channel opening. Clearly visible through the channel is the Zn(II) ion in the active site. Times shown in the figure are relative to an arbitrary start frame once equilibrium dynamics were reached, 0 ps does not represent the start of simulation.

The trajectories obtained for WT Chd revealed interesting dynamics related to substrate access. Occurring several times throughout the simulation timescale, the “right” substrate channel opens to >12 Å, which is wide enough to allow TPN to enter. After 1200 ps the “right” substrate channel closes (<8 Å), blocking TPN, but, concomitantly, the “left” channel opens (>12.5 Å), allowing TPN access to the “left” active site Zn(II) ion. Next, the “right” channel reopens, likely to allow the product 4-OH-TPN to leave the active site, but the reopening of the “right” channel concomitantly drives the “left” channel to close (<8 Å) (Figure 3, Video S2). Remarkably, coupled to the substrate channels alternately opening and closing, the corresponding proposed Cl^−^ channel opens and closes, albeit on a dramatically faster timescale (<100 ps) (Video S3). This “flip flop” mechanism indicates an allosteric effect where the two monomers in the dimer work together to direct substrate to one active site or the other and to eject Cl^−^ through a second hydrophilic channel. When observed at different points during dynamic simulations, the number of “flip-flops” was variable and the dynamic channel-opening behavior was not observed constantly. Typically, WT Chd exhibited a stable (backbone RMSD ≤ 2 Å) conformation where the only major dynamic elements appeared to be the hydrophilic heads of the helices that form the “wings” of each monomer (visible above and below the circled channels in Figure 1B), particularly surrounding Lys287 and Glu291. Curiously, in all of the TPN-bound ensembles, these dynamics were not observed, suggesting that substrate binding halted the ability of the protein to bind additional substrate.

**Figure 4:**
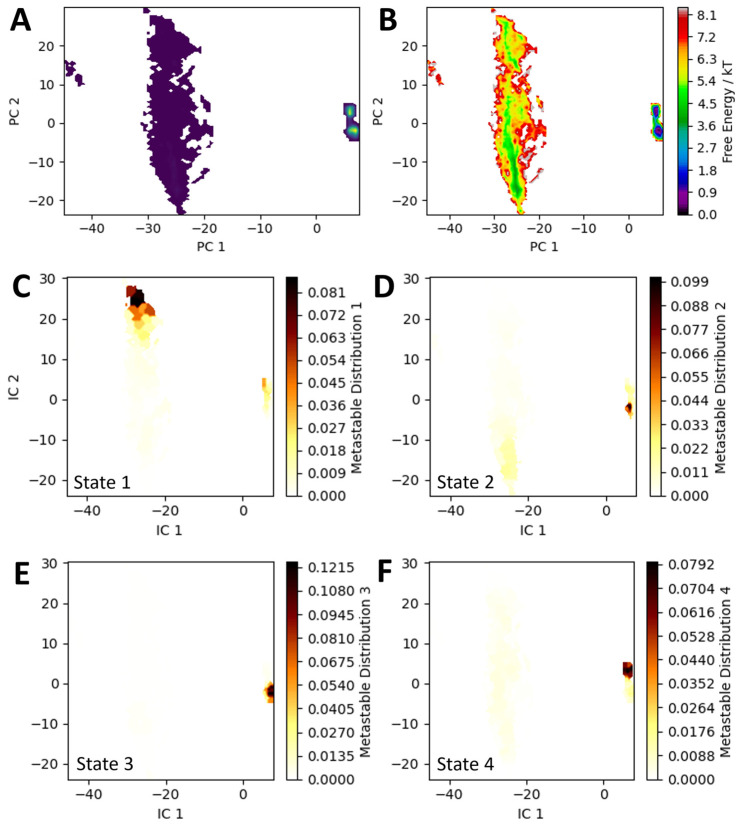
(**A**) **Representation of the WT Chd ensemble showing density within the PC space**. (**B**) Free energy of the conformations. (**C**–**F**): Metastable states 1–4 mapped onto PC space.

To quantify these dynamics, Markov state models (MSMs) were generated for all ensembles. Fortuitously, principal component analysis (PCA) for all ensembles resulted in a dimensionally reduced space with features largely parallel to the PC axes, allowing structural differences resulting from movement in PC space to be easily visualized (Figure 4 and Figure S1). It was immediately clear that trajectories sorted along PC 2 corresponded to the opening and closing of the substrate channel, with positive PC 2 values showing the “left” channel opening and negative PC 2 values showing the “right” channel opening. Moving from the highly sampled basin at positive values of PC 1 to the sparsely sampled basin at negative values of PC 1 seemed to affect the overall protein structure, but was consistently visible in the position of the wing residues Lys287 and Glu291. In both the WT Chd and WT Chd + TPN simulations, analysis of convergent MSMs indicated the presence of four metastable states and further Perron Cluster analysis revealed the distribution of these states within PC space. Three of these states (states 2, 3, and 4) lay in the highly condensed basin at positive PC 1 values and roughly neutral PC 2 values, while state 1 lay in the basin at negative PC 1 values, and either highly positive or highly negative PC 2 values, corresponding to the substrate channel being open on the “left” or “right” side, respectively (Figure 4). For WT Chd, the stationary probabilities of these states were as follows: 1: 2.1%, 2: 27%, 3: 26%, 4: 45%.

The MSM was also analyzed to find the mean first passage time (MFPT) and transition path theory (TPT) was used to determine the fluxes between metastable states. In the case of WT Chd, TPT revealed only two notable state pathways with the following layout and fluxes: 1 → 4: 98% and 1 → 2 → 4: 2%. The MFPT for the 1 → 4 transition was calculated at about 1780 ps, nearly identical to the visually observed timescale for channel opening events (~1600 ps), while the MFPT for the 1 → 2 → 4 transition is about 17,800 ps, almost exactly an order of magnitude larger than the 1 → 4 transition (Figure S2). Also of note is the relatively fast (4800 ps) 3 → 2 flux. Because states 2 and 3 have nearly the same free energy, it can be surmised that the rotation of “wing” residues are not a thermodynamically driven process, but nonetheless contribute meaningfully to Chd’s conformation (Figure 4). The degree to which the flux from 1 → 4 dominates kinetics despite the low stationary probability of state 1 is particularly interesting, highlighting the importance of channel opening for the operation of the enzyme. The much longer MFPTs for the protein to move from states 2, 3, or 4 to state 1, the low stationary probability of state 1, and the 5 kT difference between state 1 and the other basins, are consistent with a thermodynamically driven process wherein multiple internal allosteric motions must occur to generate a transient opening of the channel, which then rapidly closes. Interestingly, 5 kT is about the strength of a hydrogen bond, an energetic commitment that explains the slow dynamics towards state 1 and possibly the comparatively low *k_cat_* (~12 s^−1^) of the enzyme itself [37,38].

**Figure 5:**
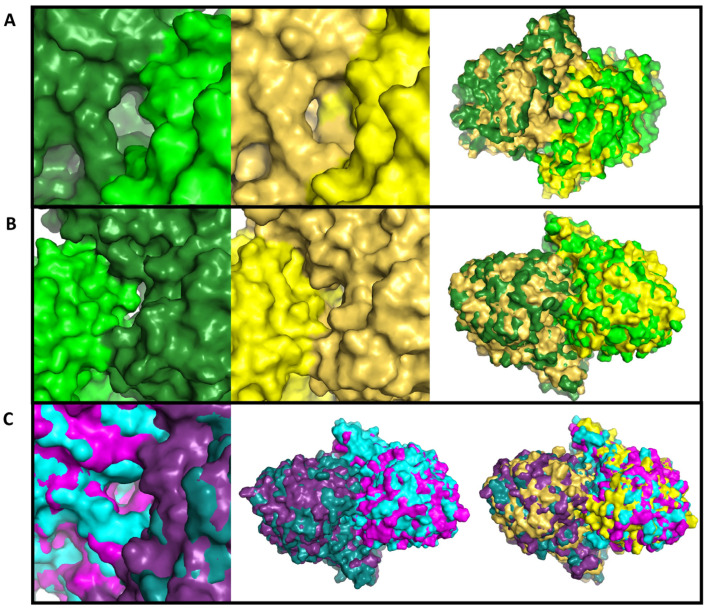
**Structural comparisons of metastable states**. For all frames, green corresponds to state 1, blue to state 2, pink to state 3, and yellow to state 4. The darker monomer is always the left monomer. (**A**) A comparison between state 1 and 4 showing the opening of the active site channel in state 1 as well as an aligned structure for both states. (**B**) Comparison between state 1 and 4 showing the open chloride channel in state 1 vs. the closed channel in state 4. Of note is the ability to see through the protein in the state 1 frame, showing the degree of dilation of both the chloride and substrate channel. Also of note is the slightly interior placement of the wing residues in state 1. (**C**) A comparison between state 2 and 3 showing the closed substrate channel and the largely identical wing residue conformation. Far right of (**C**) shows a comparison with state 4 showing that the wing residues are moved slightly interior in states 2 and 3 compared to state 4.

**Figure 6:**
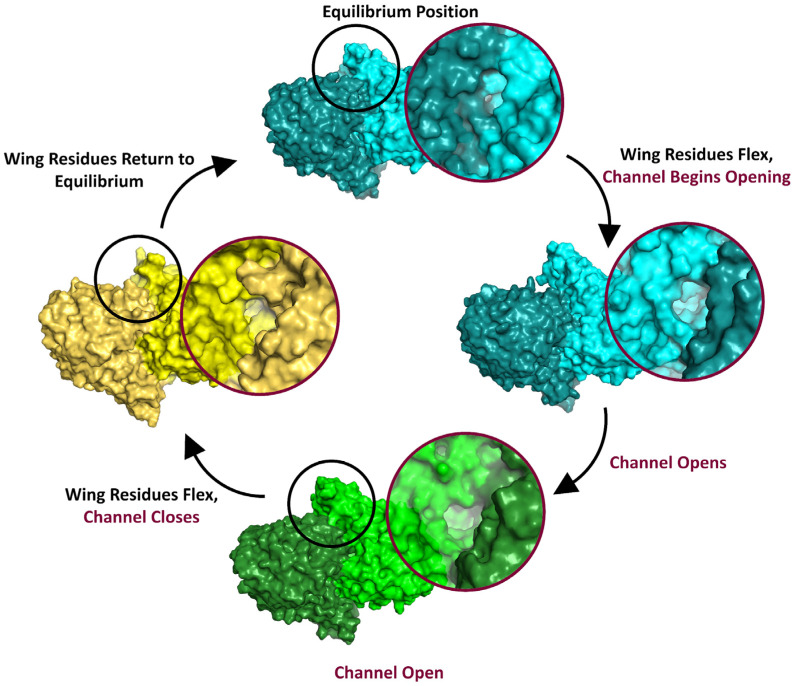
**Proposed allosteric mechanism for channel opening showing the chloride channel side of the enzyme to best visualize the wing residues.** Regions circled in black are the wing residues. Insets (circled in red) are the view down the left branch of the substrate channel. Color scheme is the same as in Figure 5. Blue corresponds to state 2, green to state 1, and yellow to state 4. The darker monomer is the left monomer.

Analysis of frames from each metastable basin serves to further show the structural changes involved in the dynamic conformations of Chd. Fifty frames were selected from each metastable basin, aligned, and compared visually. Comparisons between states 1 and 4, as expected, reveal an open substrate channel on the “left” side of Chd (Figure 5). Interestingly, state 1 always reveals a closed “right”-side chloride channel, indicating coupling of the substrate channel opening to the chloride channel closing. However, also of note is the position of the wing residues, which are consistently different between the two states. In state 1 poses, they are pulled closer to the center-of-mass of the protein and the chloride channels. This twisting motion, most apparent at the wings, is the overall motion that allows for channel opening to occur. States 2 and 3, as expected, are highly similar, and as the two states combined represent more than 50% of the trajectory, it is reasonable to assume these represent the most common dynamic state of the protein. Comparing states 2 and 3 to 4 reveal that the key differences lie in the wing residues. In both states 2 and 3, the wing residues are twisted inwards, like state 1, but not to the same degree, while in state 4 the wing residues lay further away from the protein’s center-of-mass. The MFPT from states 2, 3, and 4 to state 1 are all ~100 ns, roughly two orders of magnitude higher than the 1 → 4 transition. With these considerations in mind, the following kinetic model was proposed: the enzyme dynamically moves between states 2 and 3, begins twisting to open the left channel as signaled by the wing residues, followed by a short-lived state 1, allowing for substrate binding and channel closing. The wing residues then reverse, and the enzyme enters state 4; eventually, the enzyme relaxes back to states 2/3 (Figure 6). The last step is logical, but the length of the simulations in this work were not long enough to generate effective statistics for this relaxation, as indicated by ITS analysis during MSM construction.

**Figure 7:**
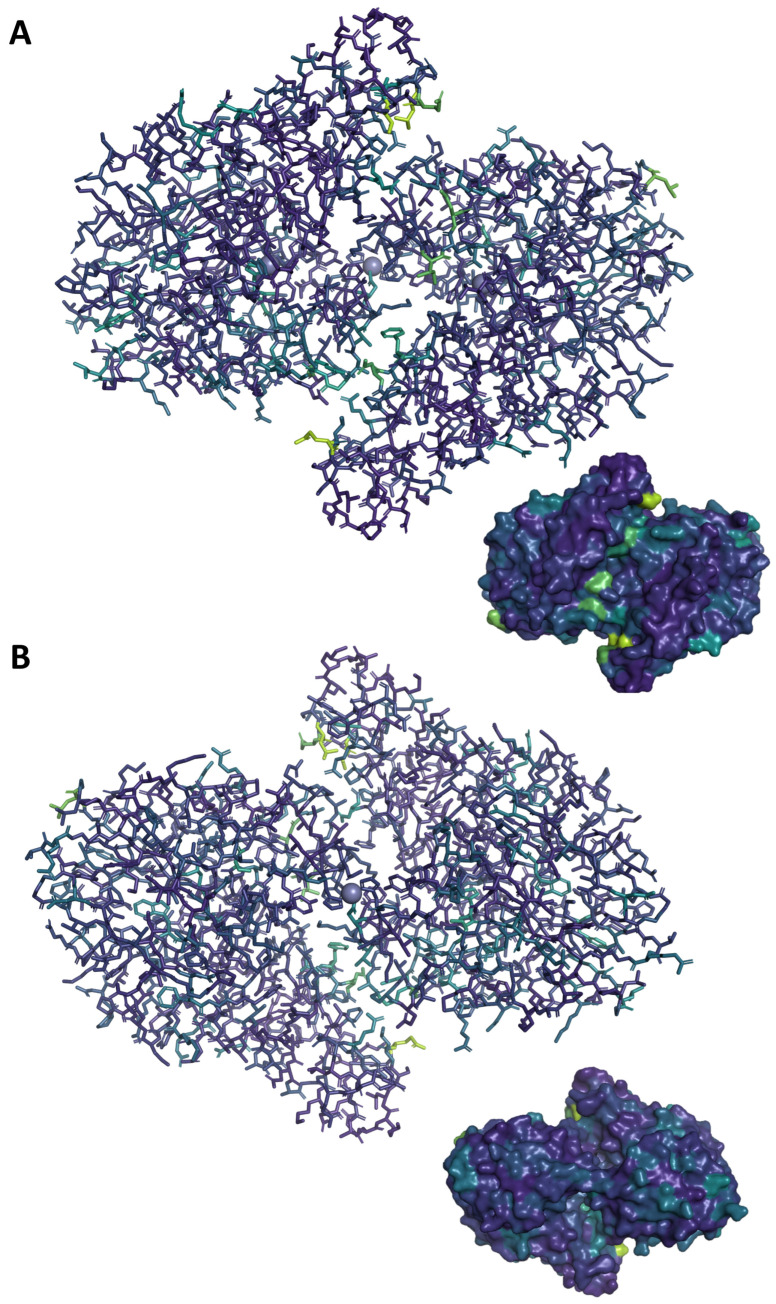
**Visualization of the BaNDyT networks shown in Figure S3 with critical nodes mapped to corresponding residues.** Of particular interest are the wing residues visible in light green, corresponding to the highest degree of allosteric importance. Also visible on both the substrate channel side (**A**) and chloride channel side (**B**) are several secondary residues dispersed through the αββα-sandwich fold common to the β-lactamase superfamily. Surface views are provided for orientation.

Two questions naturally emerge from this proposed allosteric mechanism: what significance do the wing residues have on the allosteric motion in question and what happens to the observed allostery when the substrate is bound? To address the first question, Bayesian network modeling of molecular dynamics trajectories (BaNDyT) was used to generate Bayesian network models of energy flow within Chd [34]. The wing residues, specifically Leu290, Glu291, and Val294 were immediately highlighted as allosterically important (Figure 7 and Figure S3; see Supplementary Materials for full networks). Notably, analysis of the directionality of the connections in the directed acyclic graphs (DAG) show that most of these residues accept energetic contributions from multiple interior residues, while providing energetic contributions to only a few residues, typically towards the substrate channel. This insight, coupled with the twisting motion seen in states 2 and 3, suggests a whip-like mechanism, where the necessary energy to open the substrate channel is funneled into the wing residues where they “flick” like a whip. As they relax, this indicates the enzyme is relaxing back to a state where the channels are closed. In this way, the wing residues are allosteric indicators of the flexion required for channel opening.

**Figure 8:**
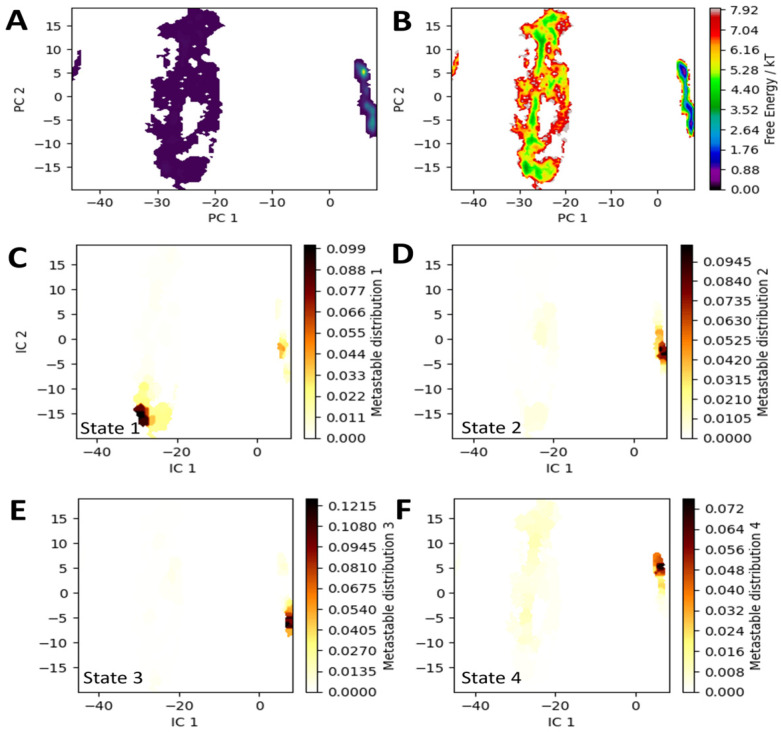
(**A**) **Representation of the WT Chd + TPN ensemble showing density within the PC space**. (**B**) Free energy of the conformations. (**C**–**F**): Metastable states 1–4 mapped onto PC space.

To address the second question, the same simulation and analysis methodology was applied to an ensemble with TPN bound in the left active site, as if it had been allowed to bind during a channel opening. As described above, the PC space for the TPN-bound ensembles were very similar, and the convergent MSM indicating highly similar structural features to the WT Chd ensemble (Figure 8). For WT Chd + TPN, the stationary probabilities of these states were also very similar: 1: 2.8%, 2: 25%, 3: 24%, 4: 49%. The conformations sampled in the metastable basins are also highly analogous, with the notable exception of state 1. In fact, the all-heavy-atom RMSD between frames taken from state 2 and 3 in the WT Chd simulation and frames taken from state 2 and 3 in the WT Chd + TPN simulation averaged only 1.5 Å. State 4, interestingly, revealed a slightly larger opening for the “left”-side channel because of TPN’s physical presence in the active site. State 1, as expected from the area it occupies in PC space, is a state where the “right” channel is open (Video S4). Of particular interest is the lack of a metastable state in the PC space corresponding to a “left”-side channel opening, indicating that, while substrate is bound in the left active site, that channel is effectively closed. Additionally, the fluxes and MFPTs governing the kinetics of these processes change significantly. Four significant fluxes were identified: 1 → 4: 42%, 1 → 2 → 4: 43%, 1 → 2 → 3 → 4: 8.2%, and 1 → 3 → 4: 6.2%. The first two fluxes are both observed in the WT Chd simulations, while the other two are not observed to be significant. Critically, all these fluxes are processes that allow relaxation after channel opening by ending at state 4. The ability of the enzyme to transition through state 2 to get to state 4 while TPN is bound may be the result of the movement of state 4 towards positive PC 2 values in the WT Chd + TPN ensembles. Therefore, it is likely that the enzyme must move through the “both channels closed” state to reach full relaxation after “right” side channel opening, although this motion need not happen linearly in PC space.

Also of interest are the differences in MFPT. The 3 → 4 MFPT remains nearly unchanged, which is consistent with the lack of an energy barrier between these states. However, the 1 → 4 transition is an entire order of magnitude slower (~20,000 ps), while the 1 → 2 transition is twice as fast (~6400 ps in WT Chd + TPN vs. ~10,000 in WT Chd). This makes the 1 → 2 → 4 MFPT ~30,000 ps, so on the same order of magnitude as the 1 → 4 transition (Figure S2). All of this is consistent with the changes in flux, where both pathways are sampled roughly equally, as opposed to a dramatic preference of the enzyme for the 1 → 4 transition in WT Chd when that transition is an order of magnitude faster than the 1 → 2 → 4 transition. Interestingly, the difference in free energy between the stable basins of states 4, 2, and 3 and the transient channel opening in state 1 is 4 kT, roughly 1 kT lower than in the WT Chd ensembles. This, coupled with the marginally higher stationary probability of state 1 in the WT Chd + TPN ensembles, could possibly indicate a positive allosteric effect wherein binding the first equivalent of TPN makes binding the second equivalent faster.

Finally, a BaNDyT network was generated for WT Chd + TPN ensembles to determine changes in allosterically important residues. The wing residues, for example, Gln292, remained important allosteric indicators of protein motion. Additionally, identical residues (e.g., Asp271 and Phe193) or nearby residues (e.g., Asp31 vs. Val35) in the same areas of the protein remained of secondary importance. Curiously, buried areas near the active site, as typified in this case by the Ile80 residue present in the left monomer, were dramatically more important energetically once TPN was bound in the active site. This is likely the mechanism by which TPN binding signals allosterically to the rest of the protein, as highlighted by allosteric connections from Ile80 to wing residues like Gln292 through other allosterically receptive residues like Leu88 (See Supplementary Materials for full networks).

## 4. Conclusions

Molecular dynamics simulations of WT Chd and WT Chd + TPN revealed a reciprocal mechanism where one substrate channel opens, i.e., “left” while the “right” channel is closed (Figure 6). Substrate association likely occurs in the common hydrophobic channel and substrate enters the “left” channel. The “right” channel then opens, likely to allow the product, 4-OH-TPN, to leave the active site, but this reopening of the right channel drives the “left” channel to close. Coupled to the substrate channels alternately opening and closing, a corresponding possible Cl^−^ channel opens and closes. This “flip flop” mechanism is indicative of the equilibrium dynamics of Chd as the enzyme must overcome a thermodynamic barrier to substrate channel opening, which requires a twisting motion of the entire dimer, as signaled by the wing residues, particularly Leu290, Glu291, and Val294. Once this twist has been generated, the enzyme affects a low population (roughly 2% of conformations) opening of the substrate channel, which closes quickly, typically in around 1700 ps. If substrate is bound during this time, the dynamics change. The active site with bound substrate is no longer able to affect channel opening. Instead, the enzyme must repeat the motion to affect opening of the opposite channel to bind additional substrate. Overcoming the energetic barrier to binding the second equivalent of substrate is marginally easier (5 kT vs. 4 kT), suggesting a mild positive allosteric effect during substrate binding. Product egress happens in much the same way, as the chloride channels are coupled to the substrate channels, indicating that a transient opening motion allows for both the hydrophobic 4-OH-TPN product and the hydrophilic Cl^−^ product to exit via separate channels tuned to their electrostatics. Future kinetic studies on an enzyme with one monomer disabled, either by genetic modification or a covalently binding inhibitor, may be able to provide experimental confirmation of these computational results.

## Data Availability

The datasets used and/or analyzed during the current study are available from the corresponding authors upon reasonable request.

## Supplementary Materials

The following supporting information can be downloaded at: https://www.mdpi.com/article/10.3390/biology15010020/s1. Figure S1: Comparison of two-dimensional principal component analysis (PCA) vs. time-lagged independent component analysis (TICA) for the WT Chd ensembles; Figure S2: Visualizations of the MFPTs between each of the four metastable states for the WT Chd; Figure S3: Visualizations of the full BaNDyT networks for WT Chd; Figure S4: Visualization showing the channel region where residue center-of-mass (COM) was added to the feature space for Markov state model (MSM) analysis; Figure S5: Visualization of the relative entropy between the WT Chd and TPN-bound Chd ensembles mapped onto one monomer with the other monomer unmapped for comparison; Figure S6: Eigenvalues for the 12 PCs required to explain 95% of the sample variance; Video S1: Overview of the structure of the Chd dimer (PDB: 6UXU), highlighting the three Zn(II) centers; Video S2: A 3000 ps visualization of the “Y-shaped” substrate channels of Chd showing the first allosteric mode observed; Video S3: 3000 ps visualization of the both the substrate channel and chloride channel of Chd; Chd is surface-colored by residue hydrophobicity (red → hydrophobic); Video S4: A 3500 ps visualization of the right side of the “Y-shaped” substrate channel of Chd showing this channel opening with altered dynamics once TPN is bound in the left active site.

## Abbreviations

The following abbreviations are used in this manuscript:

Chd	chlorothalonil dehalogenase
TPN	2,4,5,6-tetrachloroisophthalonitrile
4-OH-TPN	4-hydroxytrichloroisophthalonitrile
MBL	metallo-β-lactamase
TBP	trigonal bipyramid
MD	molecular dynamics simulations
MSM	Markov state model
COM	centers of mass
PCA	principal component analysis
TICA	time-lagged independent component analysis
ITS	Implied timescales
PCCA++	Perron Cluster
MFPT	Mean first passage time
TPT	transition path theory
BaNDyT	Bayesian network modeling of molecular dynamics trajectories
DAG	directed acyclic graphs
