# Supplementary material for: Allostery-Driven Substrate Gating in the Chlorothalonil Dehalogenase from Pseudomonas sp. CTN-3
Source: Biology (Basel). 2025 Dec 22;15(1):20. doi: 10.3390/biology15010020 (PMC12784990; doi:10.3390/biology15010020)
Supplement: Supplementary file 1 [file biology-15-00020-s001.zip › Supplemental Files/AllosterySI-For submission.docx]

**Supplemental Information for: Allostery Governs Substrate Access for the Chlorothalonil Dehalogenase from *Pseudomonas* sp. CTN-3**

Grayson Gerlich,^1,2^ Judith Klein-Seetharaman,^3*^ and Richard C. Holz,^2*^

*Contribution from the Quantitative Biosciences and Engineering Program, Colorado School of Mines, Golden, Colorado, 80401,* ^2^*Department of Chemistry, Colorado School of Mines, Golden, Colorado 80401, ^3^School of Molecular Sciences, Arizona State University, Phoenix Arizona 85004*

To whom correspondence should be addressed: Richard C. Holz, Department of Chemistry, Colorado School of Mines, Golden, Colorado 80401, Phone (303) 273-3003, E-mail: [rholz@mines.edu](mailto:rholz@mines.edu)

**Files and Descriptions**

1. PyEMMA Environment Packages.txt: plaintext file containing a list of all of the Python packages and versions necessary to replicate the PyEMMA analysis undertaken in this work.
2. PENSA Environment Packages.txt: plaintext file containing a list of all of the Python packages and versions necessary to replicate the PENSA, BaNDyT, and MDAnalysis analyses undertaken in this work.
3. WTchd_bandyt.cys: Cytoscape session file containing the full BaNDyT generated allosteric network for the WT Chd ensemble
4. WTchdtpn_bandyt.cys: Cytoscape session file containing the full BaNDyT generated allosteric network for the WT Chd + TPN ensemble.

**Video Captions**

**Video S1:** overviewvid.mp4: Overview of the structure of the Chd dimer (PDB: 6UXU), highlighting the three Zn(II) centers. Active site residues are represented by sticks, Zn(II) atoms are represented by grey spheres. Residues are otherwise colored by increasing hydrophobicity (red -> hydrophobic). The video first shows the substrate entry channel, looking down both of the channel’s “branches” at the active sites. Then, the protein is flipped to show the structural Zn(II) site, and then the chloride channels.

**Video S2:** channelswitchingvideo.mp4: A 3000 ps visualization of the “Y-shaped” substrate channels of Chd showing the first allosteric mode observed. Chd is surface colored by residue hydrophobicity (red -> hydrophobic). The distances between Asn154 and Ile29 from both chains are visualized. Clearly visible as well is the hydrophobic nature of the channel opening, which is ringed by Ile29, Phe310, and Leu307 from each chain. The “right” channel opens first, and then closes as the “left” channel opens. Towards the end of the video, the “right” channel opens again, driving the “left” channel to close.

**Video 3:** ionchannelvid.mp4: 3000 ps visualization of the both the substrate channel and chloride channel of Chd. Chd is surface colored by residue hydrophobicity (red -> hydrophobic). The allosteric connection between dilation of the substrate channel and dilation of the chloride channel is clearly visible. The chloride channel opens and closes about 5 times as quickly as the substrate channel.

**Video 4:** tpnboundvid.mp4: A 3500 ps visualization of the right side of the “Y-shaped” substrate channel of Chd showing this channel opening with altered dynamics once TPN is bound in the left active site. Chd is surface colored by residue hydrophobicity (red -> hydrophobic). The distance between Asn154 and Ile29 is visualized. TPN is visible in green. Channel opening is much slower in this mode, but channel closing is still fast.

**Figures**

**
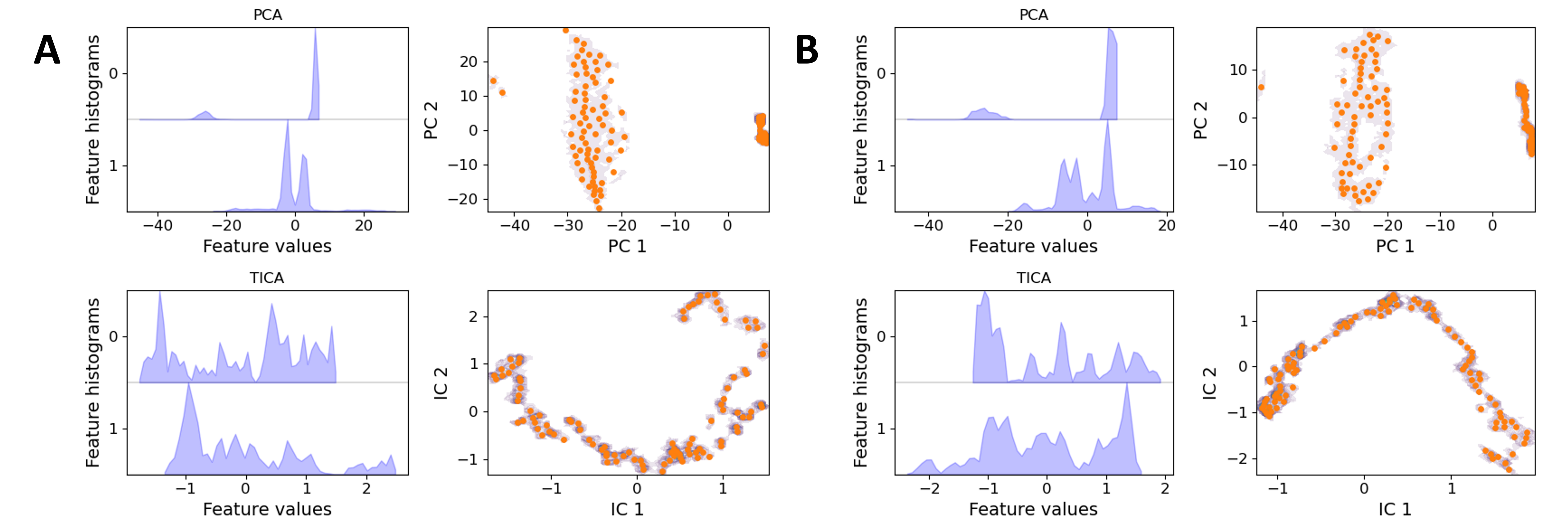
**

**Figure S1.** *Comparison of two-dimensional principal component analysis (PCA) vs time-lagged independent component analysis (TICA) for the WT Chd ensembles.* Orange dots represent clusters, with 100 clusters for both approaches. The globular clustering in the PCA plot, with the main features parallel with a component axis, was easier to parse and was less prone to generating MSM’s with disjoint states. The added benefit of clusters parallel to principle components was the ability to visualize frames along one component at a static place in the second to identify complex motions associated with each component. **A** shows the clustering for WT Chd, and **B** shows the clustering for WT Chd + TPN. The PCA spaces of the two substrate bound simulations were nearly indistinguishable.

**
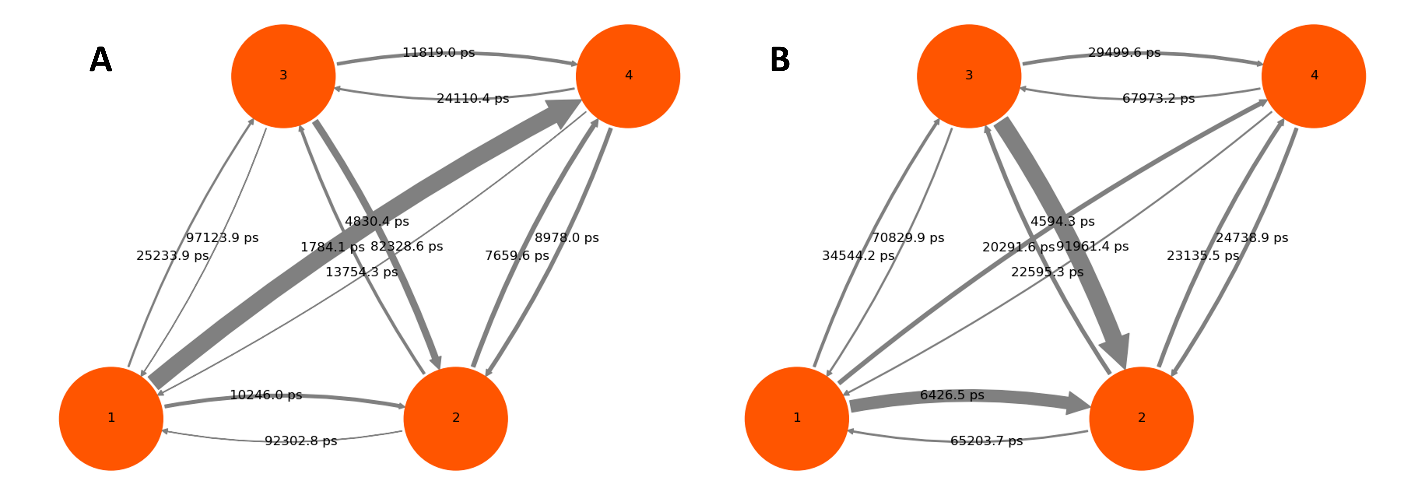
**

**Figure S2.** *Visualizations of the MFPTs between each of the four metastable states for the WT Chd*. (**A**) and WT Chd + TPN (**B**) simulations. Arrow weights are larger for faster (therefore better sampled) processes, while MFPTs are listed over each flux in the DAG in ps.


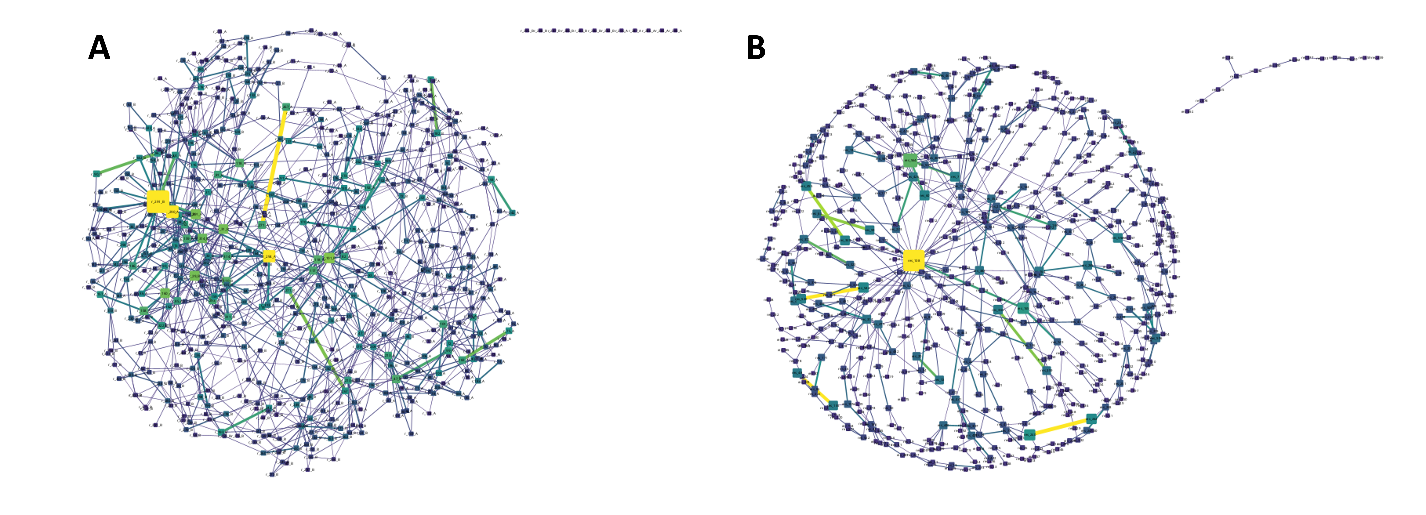


**Figure S3.** *Visualizations of the full BaNDyT networks for WT Chd.* (**A**) and WT Chd + TPN (**B**) simulations. As these are challenging to view at this scale, these images are provided for context only. The full networks are provided as Cytoscape .cys files in the SI.

**
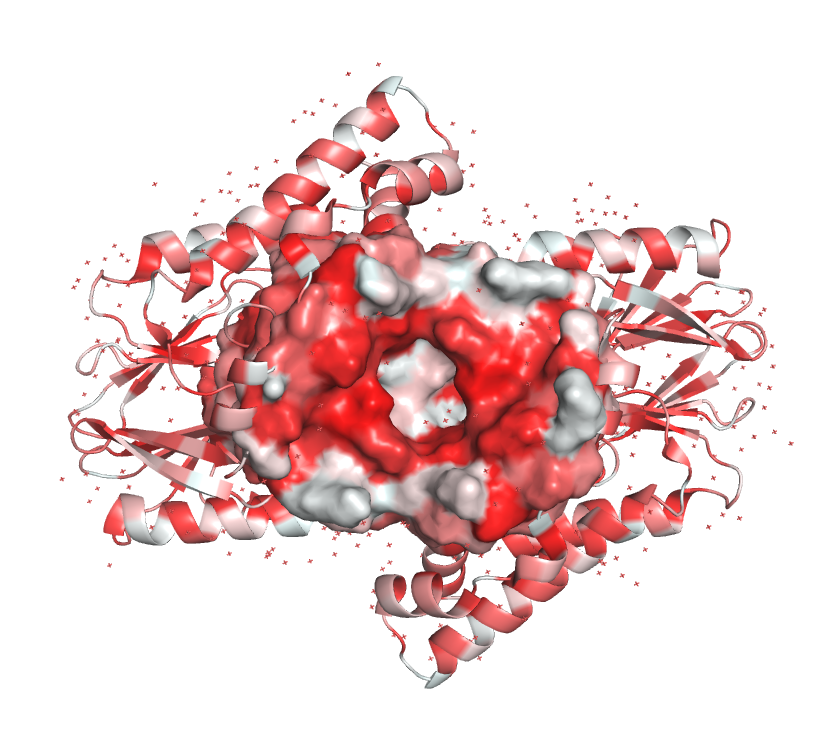
**

**Figure S4.** *Visualization showing the channel region where residue center of mass (COM) was added to the feature space for Markov state model (MSM) analysis.* The full Chd structure (PDB: 6UXU) is visualized as a cartoon, while the channel region is visualized as a surface. All residues are colored by hydrophobicity (red -> hydrophobic).

**
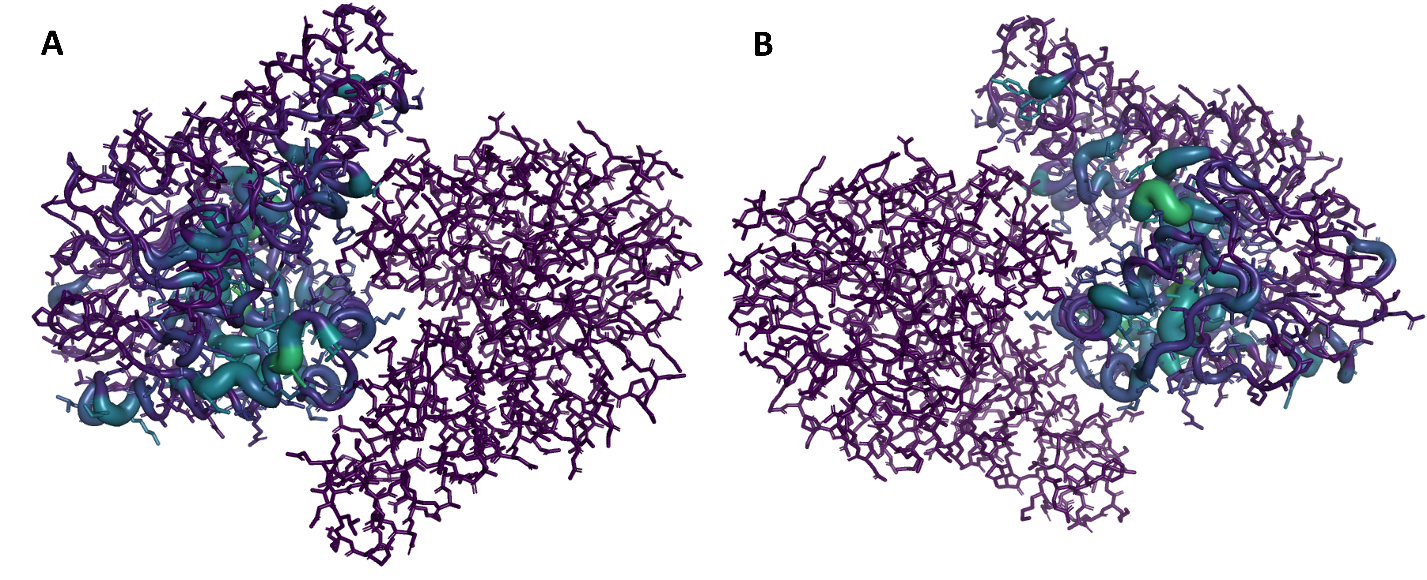
**

**Figure S5.** *Visualization of the relative entropy between the WT Chd and TPN-bound Chd ensembles mapped onto one monomer with the other monomer unmapped for comparison.* The color and thickness of the cartoon represents the Jenson-Shannon distance (using the standard viridis color scheme). The channel region encompasses most of these residues, so the key COMs are covered in the MSM analysis. **A** shows the substrate channel side, **B** shows the chloride channel side.

**
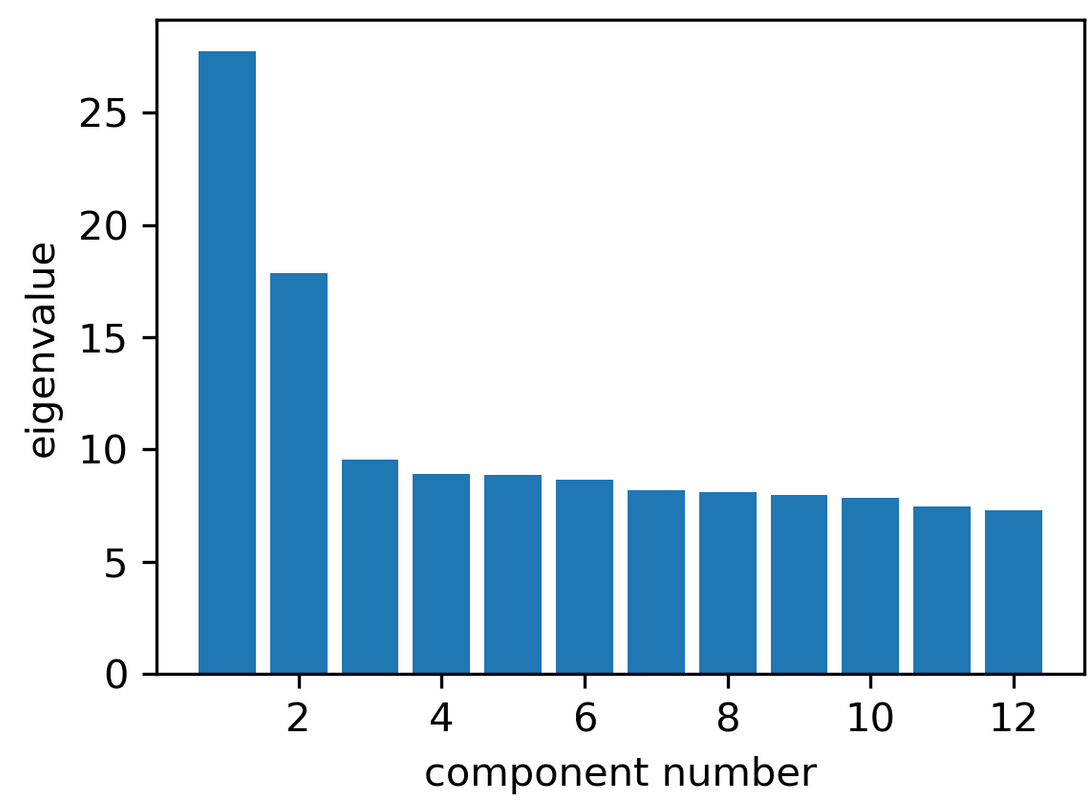
**

**Figure S6.** Eigenvalues for the 12 PCs required to explain 95% of the sample variance.
