# Supplementary figures and images for: Allostery-Driven Substrate Gating in the Chlorothalonil Dehalogenase from Pseudomonas sp. CTN-3
Source: Biology (Basel). 2025 Dec 22;15(1):20. doi: 10.3390/biology15010020 (PMC12784990; doi:10.3390/biology15010020)

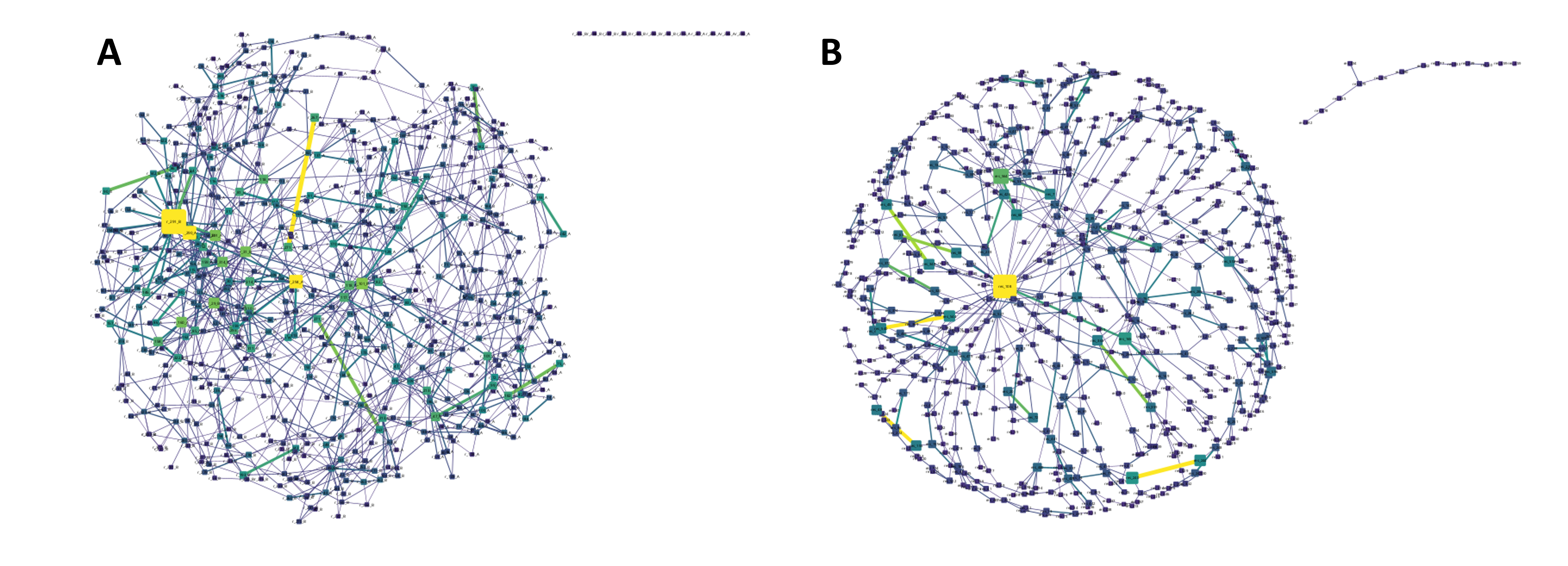

Supplement: Supplementary file 1 [file biology-15-00020-s001.zip › Supplemental Files/SIFig6.png]

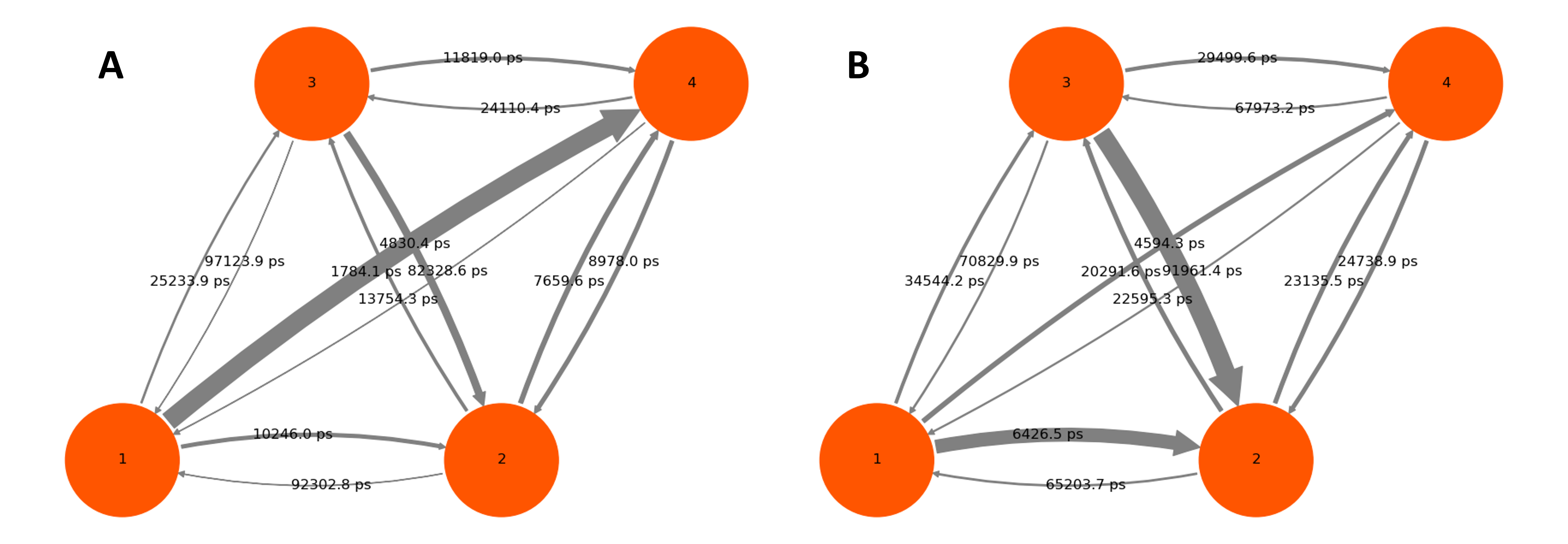

Supplement: Supplementary file 1 [file biology-15-00020-s001.zip › Supplemental Files/SIFig5.png]

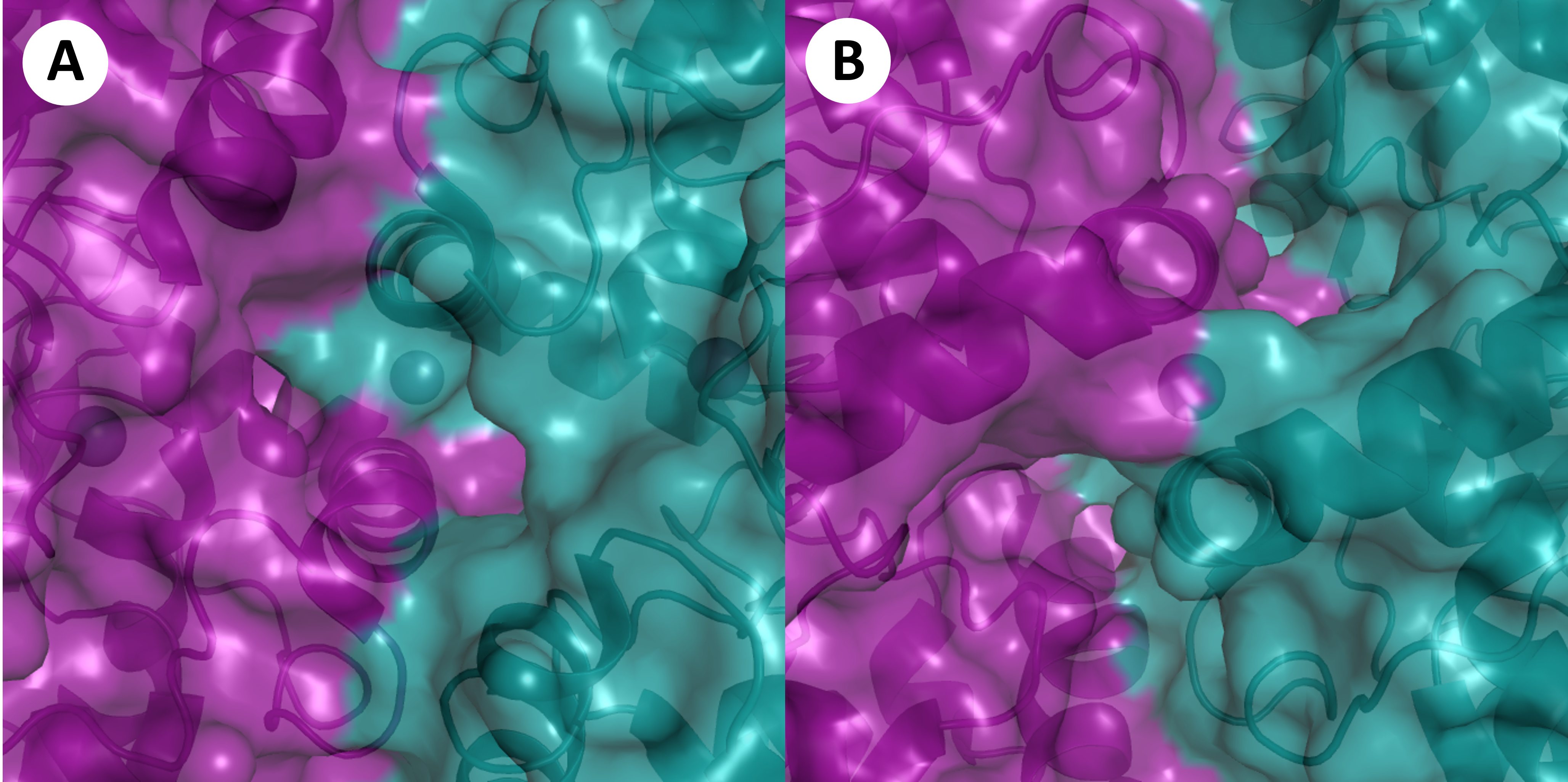

Supplement: Supplementary file 1 [file biology-15-00020-s001.zip › Supplemental Files/SIFig4.png]

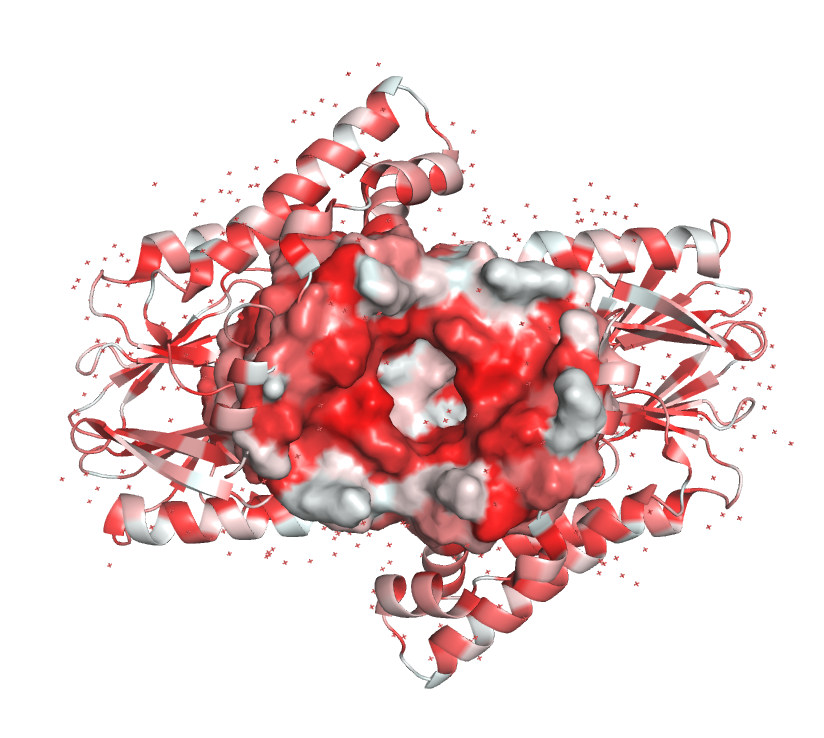

Supplement: Supplementary file 1 [file biology-15-00020-s001.zip › Supplemental Files/SIFig1.png]

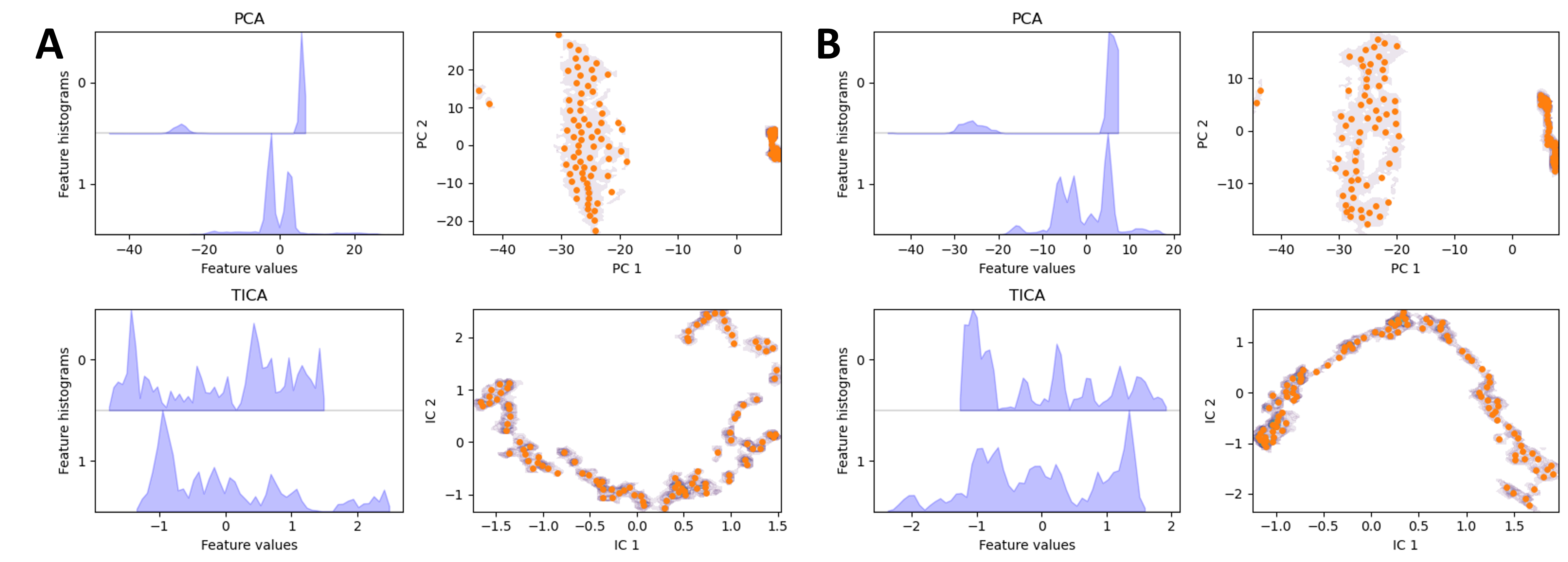

Supplement: Supplementary file 1 [file biology-15-00020-s001.zip › Supplemental Files/SIFig3.png]

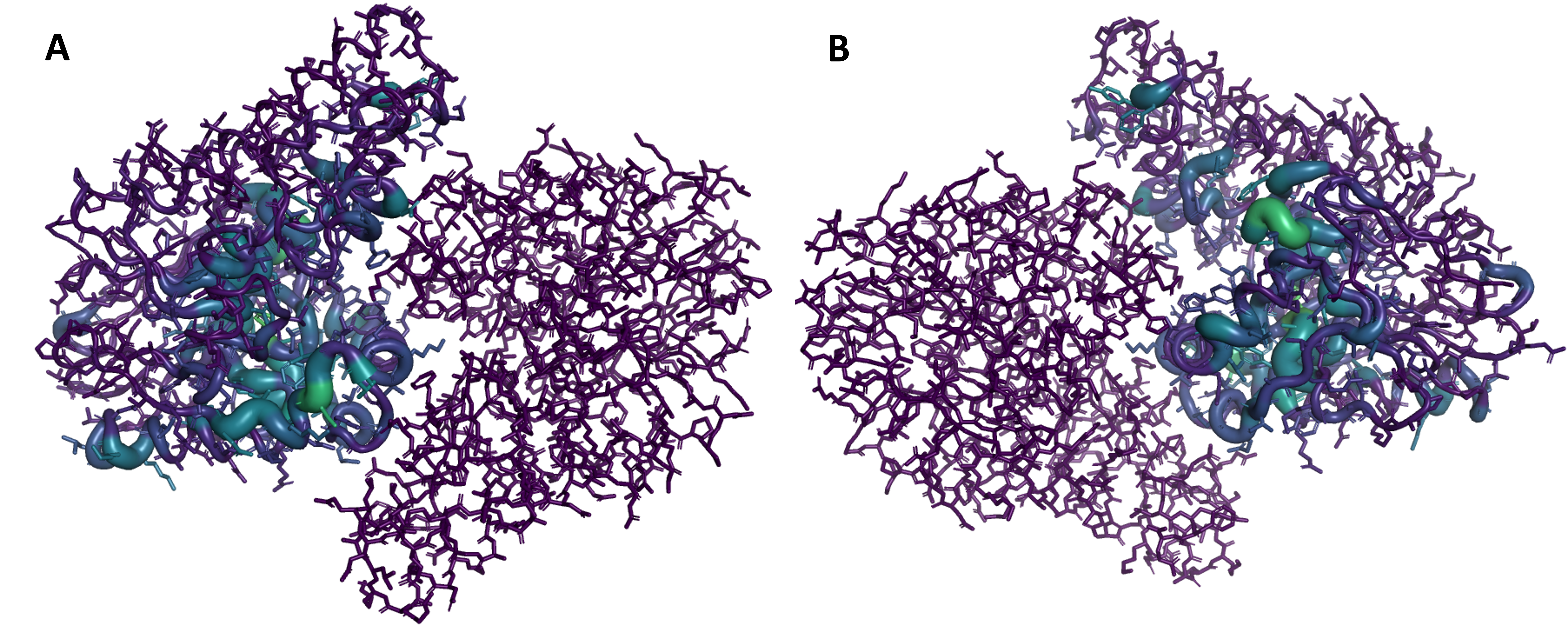

Supplement: Supplementary file 1 [file biology-15-00020-s001.zip › Supplemental Files/SIFig2.png]
